# Descriptive study of goat external and middle ear through computed tomography and endoscopic evaluation, compared with the human ear

**DOI:** 10.1016/j.bjorl.2021.07.006

**Published:** 2021-10-13

**Authors:** Larissa Leal Coutinho, Pâmella Marletti de Barros, Mariana de Carvalho Leal, Silvio da Silva Caldas Neto, Thiago Freire Pinto Bezerra

**Affiliations:** Universidade Federal de Pernambuco, Recife, PE, Brazil

**Keywords:** External ear, Middle ear, Goats, Computed tomography

## Abstract

**Objective:**

To describe the external and middle ear of goats, using computed tomography and endoscopic dissection, comparing them with the anatomy of the human ear, aiming to evaluate the viability of this animal model for training in otological endoscopic surgery.

**Methods:**

Images of the external and middle ear of goats were produced by computed tomography in the coronal and axial planes and analyzed. The radiological anatomy of the external and middle goat and human ear are described. Some measures of surgical interest were described, such as the length of the external auditory canal, external and internal cross-sectional area of the external auditory canal, middle ear depth, vertical angle of the external auditory canal, length of malleus and incus. After the tomographic study, we analyzed the importance of evaluations through the endoscopic dissection of the goat ear.

**Results:**

The bony canal is extremely tortuous and shows a bony prominence with significant elevation near the lower edge of the tympanic membrane. The middle ear has a large tympanic bulla in the hypotympanum region. The malleus is shaped similarly to that of the human, but its handle is located anterior to its head. The mastoid is not pneumatized. Compared to the human mastoid, there was a statistically significant difference in the external and internal cross-sectional area, at the depth of the middle ear, in the vertical angle of the canal and in the length of the incus.

**Conclusion:**

Goat morphometric anatomy showed that this model is suitable for surgical simulation in the initial training for endoscopic otological surgery. It is an important contribution to the development of skills to accelerate the subsequent endoscopic otological surgical training in humans, despite some differences found.

## Highlights

• The goat ear anatomy model is highly accessible in developing countries.
• The goat's external auditory canal is tortuous and long.
• The goat bone canal has a bony prominence in its lower portion.
• It is necessary to drill the bone prominence to visualize the entire tympanic membrane.
• The goat model allows training with an endoscope and otological surgery steps.

## Introduction

Mer, in 1967, was the first to describe the use of an endoscope to visualize the anatomy of the middle ear (ME).^1^ After two decades, Nomura promoted the idea of ​​a myringotomy in an intact tympanic membrane to allow the endoscopic examination of human ME structures.^2^ The endoscope offers a wider visualization, in addition to being less invasive, without requiring retroauricular incisions. Also, in terms of visualization, it becomes easier to see locations that are difficult to access, such as the epitympanic spaces, tympanic sinus and hypotympanum. Other advantages include shorter operative time, faster recovery, and less postoperative pain.^3^, ^4^, ^5^, ^6^

Surgical simulation is a valuable part of surgical education, with a beneficial effect on surgeon competence and patient safety.

The training of surgical techniques on cadavers plays an important role in the surgeon’s training and improvement, in addition to providing the best anatomical accuracy. However, increasing costs and the medical-legal issues have made cadaver ear availability increasingly more difficult.

Additional training tools for surgeons are synthetic and virtual simulators which, despite having good anatomical fidelity and realistic sensation of the surgical act, have a high cost, hindering the dissemination of their use.

The growing need for alternatives to improve skills in otological surgery resulted in animal studies to validate models for training, always aiming at the condition that the more similar to the human being, the better.

Lavinsky and Goycoolea described sheep as a possible animal model for otological surgery.^7^

Another study showed that the sheep middle ear had many similarities when compared to the human one and could be used in the training of surgical techniques for ossicular chain reconstruction.^8^

Anschuetz et al. developed a surgical training program for canaloplasty, myringoplasty, and ossiculoplasty and validated an *ex vivo* animal model for endoscopic otological surgery exclusively in sheep.^9^

Zaidi et al. described that the goat middle ear would be similar to a human one. However, this study showed some important differences, such as the more anterior position of the malleus, a very large and voluminous tensor tympani muscle present medially, while the stapes has a different orientation compared to the human stapes and the facial nerve is protected by a bony prominence. The differences found associated with the existence of a small sample of studied specimens highlighted the need for further studies to confirm these findings.^10^

The *Capra aegagrus hircus* and sheep are from the *Bovidae* family and *Caprinae* subfamily, which share anatomical similarities. Despite this, there are few publications on the use of goats as an anatomical training model in endoscopic otological surgery. Therefore, the study of the goat ear becomes relevant to aid the development of surgical skills in a surgical training model of very low cost and great accessibility in developing countries.

## Objective

Describe the external and middle ear of goats, through images obtained through computed tomography, comparing them to the human ear anatomy.

Describe the anatomical findings through endoscopic dissection of the goat's ear.

## Methods

Thirty temporal bones of Boer goats, a meat production lineage, weighing between 26 and 30 kg, and aged between 4 and 6 months, slaughtered for human consumption, were studied.

The study was submitted to the Ethics Committee on Animal Use (CEUA) and received authorization to be carried out, as it utilized animal disposal material, under Protocol N. 0040/2017.

The goat heads were acquired through donation and were frozen in a freezer, inside plastic bags, at a temperature of −18 °C.

The computed tomography images were performed using a Toshiba® tomograph, model Xvision EX, with a tube rotation time of 1 s. The examinations were carried out according to the protocol for studies of human temporal bones, with a 0.6-mm thick section.

**Figure 1:**
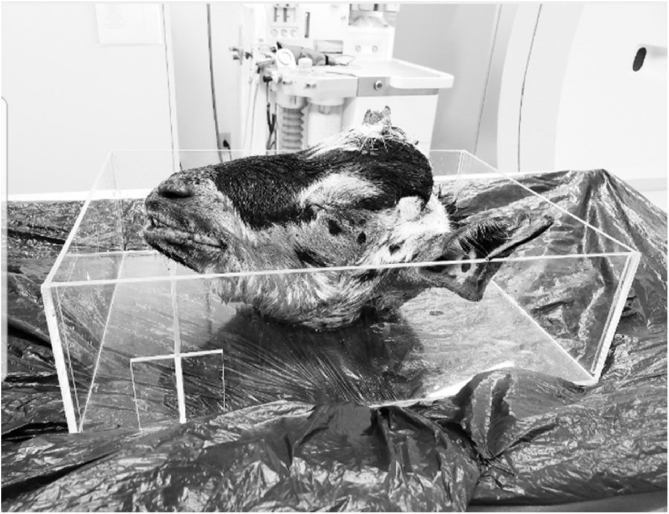
Acrylic support. Transparent acrylic support. Measurements: 50 × 30 × 15 cm.

A stabile support was developed to accommodate the animals' heads, so that they remained in a single position, ensuring precision and uniformity during image acquisition through tomographic sections (Fig. 1).

As the animals belong to the same breed and lineage and there is no great genetic variability, there was no need for sample calculation. As the study is a descriptive one, a convenience sample of 30 ears was determined.

### Measurements

The computed tomographies were stored in an external HD unit for later interpretation. The first measurements of the anatomical references were made and guided by an experienced otorhinolaryngologist. After that, the other measurements were taken together with a radiologist. Other structures such as the tympanic membrane and mastoid cavity were also evaluated and described.

**Figure 2:**
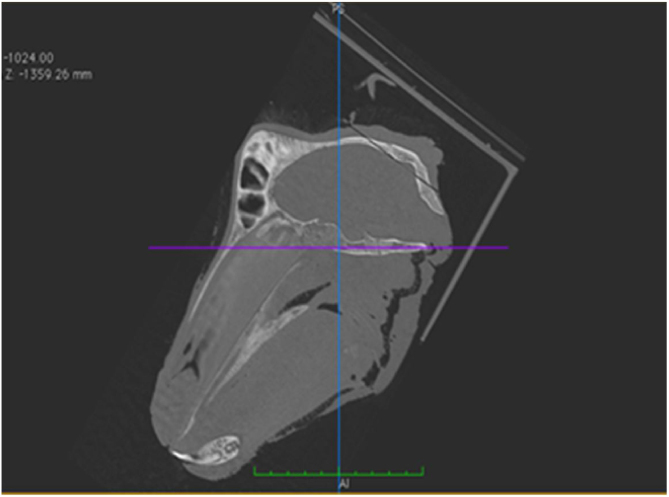
Plane used to measure the structures of the goat ear.

The horizontal plane was considered to be the skull base plane, as the orientation of the goat ear axis would be closer to that of the human ear (Fig. 2). All measurements of the goat ear described below were taken from tomographic images, using a bone window and coronal section.

**Figure 3:**
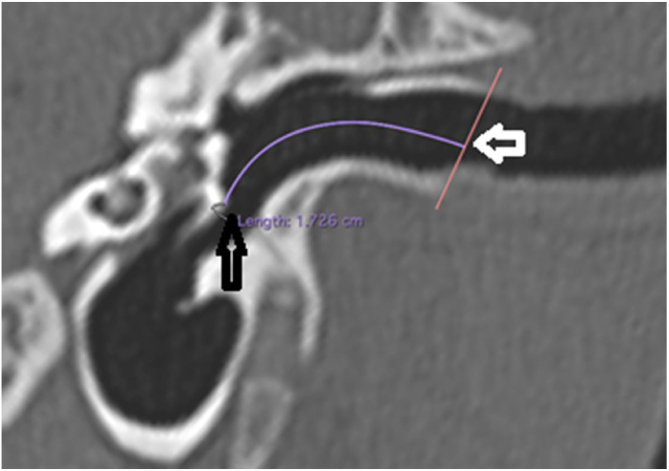
Length of the bone canal. Reconstruction in the plane of the external auditory canal, left ear. The curved line represents the length of the bone canal from the lateral process of the malleus. White arrow: lateral end of the external ear canal; black arrow: lateral process of the malleus.

The length of the bony canal (LBC) was measured in a vertical section in the canal plane, with a curved line parallel to the floor, starting from the lateral process of the malleus to the lateral edge of the external auditory canal (Fig. 3).

**Figure 4:**
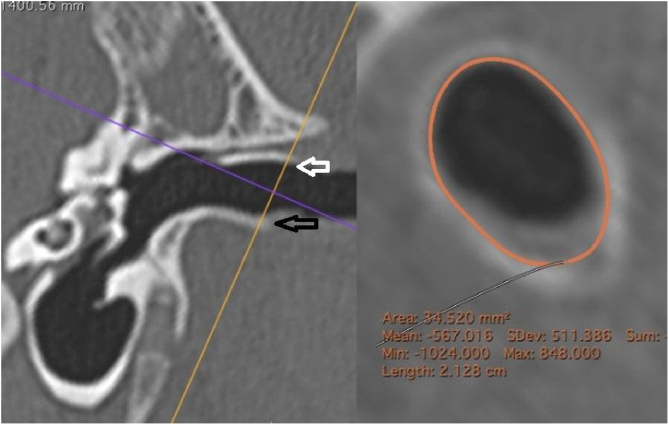
Measurement of the external area of the canal. On the left, the section line (orange) used. On the right, the reconstructed image with the calculated measurement. White arrow: upper edge of the lateral extremity of the external auditory canal; black arrow: lower edge of the lateral end of the external auditory canal.

The external cross-sectional area of ​​the canal (ECSAC) was measured using a cross-sectional plane of the lateral extremity of the bone canal (Fig. 4).

**Figure 5:**
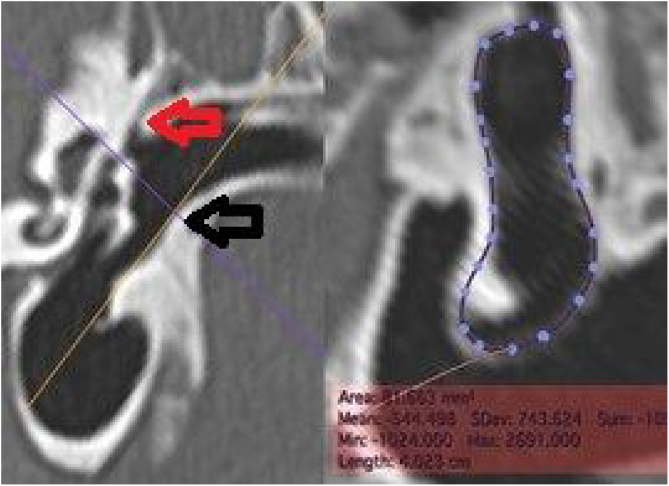
Measurement of the medial area of the canal. On the left, the section line (orange) used. On the right, the reconstructed image, with the area measurement. The dotted line represents the contour of the inner margin of the canal, where the tympanic membrane is inserted. Red arrow: upper edge of the medial extremity of the external ear canal; black arrow: lower edge of the medial extremity of the external auditory canal.

The internal cross-sectional area of ​​the anal (ICSAC) was measured using a cut at the medial extremity of the bone canal (Fig. 5).

**Figure 6:**
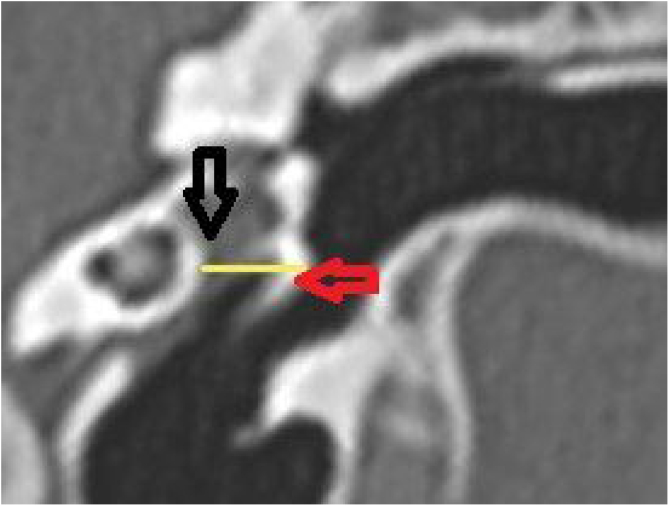
Middle ear depth measurement. Vertical section in the plane of the canal. The yellow line shows the depth of the middle ear. Red arrow: lateral process of the malleus; black arrow: medial wall of the middle ear.

The middle ear depth (MED) was measured in a horizontal plane, starting from the lateral process of the malleus to the medial wall (Fig. 6).

**Figure 7:**
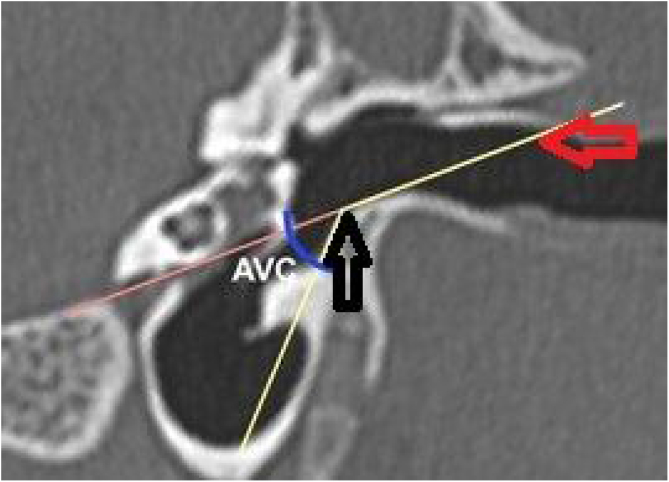
Measurement of the vertical angle of the canal. Vertical section in the plane of the canal, showing the procedures for measuring the angle of the canal. Red arrow: upper edge of the lateral extremity of the external ear canal; black arrow: floor of the external ear canal.

The vertical angle of the canal (VAC) was measured in a vertical section in the plane of the canal. Two lines were drawn. One, from the upper edge of the bone canal, was tangent to the floor where the canal curves downward. The other was tangent to the floor just after this curvature. The angle formed between these two lines was then measured (Fig. 7).

**Figure 8:**
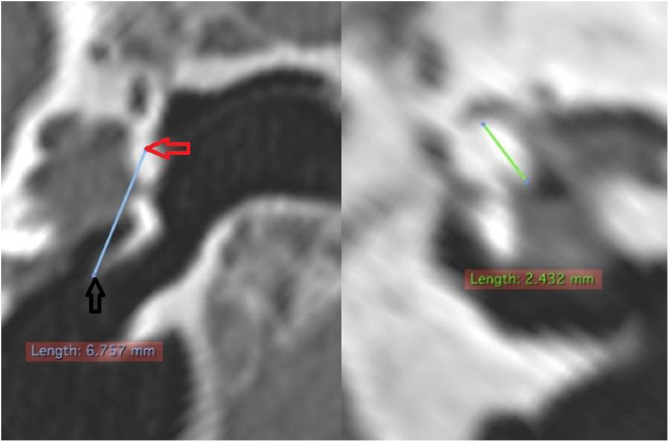
Measurement of the length of the malleus and incus. On the left, measurement of the length of the malleus. On the right, of the incus. Red arrow: upper pole of malleus head; black arrow: umbo.

The length of the malleus (LM) was measured in a reconstructed section from the upper pole of its head to the umbo. Regarding the length of the incus (LI), due to its small size, it was not always possible to identify it in tomographic images. When it was possible to identify it, its measurement was performed by searching for the best section plane to do it (Fig. 8).

**Figure 9:**
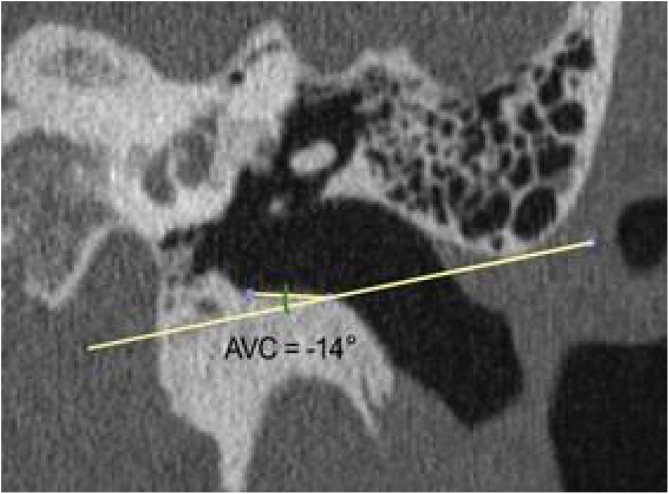
Measurement of VAC in a human subject. The measurement of the angle, in this case, was considered equal to zero.

The same measurements were obtained from computed tomography images of 30 adult human temporal bones to be used as the basis for comparison with the animals' ears. There were two differences in the methodology for obtaining measurements: 1) The length of the canal, instead of being measured from the lateral process of the malleus, was measured from the umbo of the tympanic membrane. The reason for this difference is that the umbo, in humans, represents the most central point of the membrane, while in goats, the center is located in the lateral process of the malleus; 2) The angulation of the canal in humans is less marked than in goats. Generally, by drawing a line from the upper edge of the lateral extremity of the bone canal, direct access to the inferior margin of the tympanic membrane is easily achieved. Therefore, the VAC is less than zero. As the important thing is to have access to the inferior margin, any VAC that was equal to or less than zero was considered zero (Fig. 9). Only 7 bones had a VAC greater than zero.

**Figure 10:**
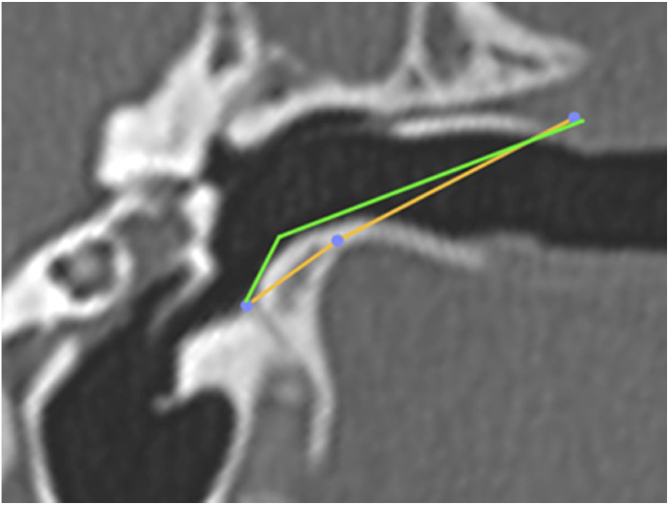
Angulation of the canal before and after drilling. The green line shows the angulation of the canal showing the trajectory tangent to the curvature of the floor. The orange line shows the angulation if the floor was drilled up to the limit of the infratemporal soft tissues. In this case, from a VAC of 51°, we would have an angle of 8°.

Subsequently, we simulated the drilling of the bone in the caprine external auditory canal floor, at the point of its inferior deflection, to reduce its angulation. This would facilitate the visualization of the ear anatomy, as well as the reach of the surgical instruments. The difference in VAC was measured before and after drilling to assess the extent of gain with this procedure (Fig. 10). The vertical angle of the canal before the simulation of the floor drilling was defined as actual VAC, and simulated VAC, the angle found after this drilling.

### Endoscopic evaluation of the goat ear

One of the animal's heads was randomly selected for endoscopic dissection. It was defrosted for 24 h in a container with a solution with vinegar, at a concentration of 15 g/L (15 g of vinegar in 1 L of water), enough to cover the entire goat head. A 4 mm optic connected to a Full HD Astus Medical video system was used for the study, in addition to microsurgical instruments for handling.

The head was placed on a fixed surface, in the surgical position. Resection of the cartilaginous portion of the external auditory canal was performed, followed by macroscopic evaluation of the bone canal and tympanic membrane; drilling of the lower portion of the BC to access the lower edge of the tympanic membrane; creation of the tympanomeatal flap on the posterior wall of the BC, followed by its detachment until the identification of the tympanic annulus and elevation of the tympanic membrane and the evaluation of the middle ear.

### Statistical tests

All goat and human ear measurements were submitted to descriptive statistical analysis. The median, interquartile range, maximum and minimum values ​​were calculated. For comparison of measurements, the Mann-Whitney-*U* test was used. A *p* value <0.05 was considered as statistically significant. The measurements of the anatomical structures of goats and humans were evaluated through graphical evaluation in Box-Plot graphs.

## Results

Thirty goat temporal bones were studied. The left temporal bone was chosen for the first evaluation. It was not possible to identify the incus in 5 goat right temporal bones and 5 goat left temporal bones, with a total n = 20 for this anatomical structure. The other structures in the animal and human models had n = 30. The measurements of the anatomical structures evaluated are described in Table 1.

**Table 1:** Measurements obtained from the 30 assessed goat temporal bones.

	LBC (mm)		ECSAC (mm^2^)		ICSAC (mm^2^)		MED (mm)		VAC (°)		LM (mm)		LI (mm)	
Animal	RE	LE	RE	LE	RE	LE	RE	LE	RE	LE	RE	LE	RE	LE
01	18.1	17	34.9	23.7	53.1	53.5	4.7	4.2	51	51	7.0	7.0	2.1	?
02	15.8	15	29.4	29	54.9	54.6	4.8	4.8	47	47	7.0	7.1	2.5	2.7
03	12.5	13	40.7	40.4	80.0	80.6	4.3	4.3	45	44	7.3	7.7	3.0	2.9
04	14.7	15.4	36.9	36.8	79.6	79.1	4.6	4.6	47	47	7.6	7.5	?	3.0
05	15.0	15.4	45.0	45.8	67.8	68	4.8	4.4	37	37	7.0	6.9	3.0	?
06	19.9	18.8	55.0	54.4	76.4	75.9	3.8	3.6	30	31	7.6	7.8	?	?
07	14.9	14.6	33.9	35.5	79.3	79.3	4.4	4.0	27	27	7.3	7.3	?	?
08	13.6	13.6	42.6	42.8	82.9	82.1	4.1	4.2	39	39	7.4	7.4	?	2.8
09	15.5	15.2	35.7	35.1	86	86.3	4.4	4.0	47	47	7.8	7.9	3.5	3.2
10	12.3	13.9	30.6	30.7	73.0	73.2	4.9	3.9	65	65	7.5	7.4	3.0	3.0
11	15.0	15.5	24.0	25.0	61.5	61.0	4.2	4.1	35	38	7.2	7.0	2.5	2.9
12	17.7	17.0	30.7	31.5	72.6	73.0	73.2	4.9	43	40	7.5	7.3	3.2	3.0
13	15.3	15.0	36.8	36.0	75.4	75.5	61.0	4.2	30	35	7.0	7.1	2.7	3.1
14	13.7	12.8	41.4	42.1	55.7	55.0	3.2	3.7	52	50	7.8	7.5	3.0	2.9
15	16.5	15.8	28.7	29.3	80.1	81.5	4.0	4.3	49	52	7.4	7.2	?	?

LBC, Length of bone canal; ECSAC, External cross-sectional area of the canal; ICSAC, Internal cross-sectional area of the canal; MED, Middle ear depth; VAC, Vertical angle of canal; LM, Length of malleus; LI, Length of incus.

The external auditory canal was tortuous and long. Further, due to this tortuosity, it was not possible to define, through tomographic images, where the external auditory canal was located.

The bony portion of the canal curves inferiorly so that the lower edge of the tympanic membrane lies at a much lower level than the plane of the rest of the canal. This is likely to prevent a direct visualization of the lower edge of the tympanic membrane. The canal invariably shows a well-developed and rounded bony prominence, close to this lower border of the tympanic membrane, which can make it even more difficult to visualize this region.

On tomographic images, it is possible to observe that the tympanic membrane is wide with a large *pars flaccida*, that has almost the same area as the *pars tensa*. The *pars flaccida* extends from the margin of the tympanic notches to the lateral process of the malleus. The *pars tensa*, on the other hand, is located caudally to the lateral process of the malleus. The upper edge of the tympanic annulus is located laterally, so that the *pars flaccida* seems, in the coronal section, to occupy a large part of the canal. As the roof of the bony canal is much shorter than the floor, the tympanic membrane takes on an oblique direction.

The middle ear consists of a large tympanic bulla, which extends inferiorly, anteriorly and posteriorly to the canal. The part of this bulla that corresponds to the tympanic membrane area represents a small number when compared to the total volume of the bulla.

Regarding the ossicular chain, the malleus and incus were visualized. It was not possible to identify the incus in 10 goat temporal bones. The stapes was not visualized. The malleus is located in a slightly different position than the human malleus does, with its handle located anterior to its head. The incus is located caudally to the malleus, in the epitympanic recess.

The mastoid of all animals was ivory-colored, without any pneumatization.

Table 2 shows the descriptive statistical measures of the goat ear.

**Table 2:** Descriptive statistical measures related to goat ear.

	LBC (mm)	ECSAC (mm^2^)	ICSAC (mm^2^)	MED (mm)	VAC (°)	LM (mm)	LI (mm)
**Median**	15,10	23,85	75,45	4,2	44,5	7,35	3,00
**Interquartile range**	2,13	12,27	18,65	0,45	13,00	0,45	0,27
**Maximum**	19,9	55,00	86,39	4,9	65,00	7,9	3,5
**Minimum**	12,30	23,70	53,10	3,20	27,00	6,9	2,10

LBC, Length of bone canal; ECSAC, External cross-sectional area of the canal; ICSAC, Internal cross-sectional area of the canal; MED, Middle ear depth; VAC, Vertical angle of canal; LM, Length of malleus; LI, Length of incus.

Table 3 shows the same measurements performed on 30 human temporal bones. The statistical analysis of these measurements is shown in Table 4.

**Table 3:** Measurements obtained from the ears of 30 adult human temporal bones.

	LBC (mm)		ECSAC (mm^2^)		ICSAC (mm^2^)		MED (mm)		VAC (°)		LM (mm)		LI (mm)	
	RE	LE	RE	LE	RE	LE	RE	LE	RE	LE	RE	LE	RE	LE
**1**	15.1	15.5	87.1	86.4	65.2	65.9	5.0	5.0	8	8	6.6	6.5	6.5	6.7
**2**	14.8	14.9	50.5	61.4	71.0	71.6	7.2	7.1	0	0	6.3	6.8	6.8	6.8
**3**	13.0	13.5	73.2	73.1	52.5	51.9	7.5	7.1	0	0	7.8	8.0	5.4	5.2
**4**	13.5	13.5	70.0	71.2	58.4	58.0	6.4	6.5	0	0	6.6	6.6	5.0	5.1
**5**	16.2	15.8	68.5	68.9	50.2	50.5	5.9	6.0	0	0	7.2	7.6	5.4	5.4
**6**	13.6	13.9	80.3	81.1	82.5	82.7	6.3	6.7	10	11	8.5	8.6	6.1	6.5
**7**	15.9	15.0	20.8	20.2	62.9	62.0	7.3	7.3	0	0	6.0	6.4	6.0	5.7
**8**	16.0	16.3	64.41	63.9	83.9	83.5	7.0	7.3	0	0	7.9	8.0	6.7	6.7
**9**	13.6	13.1	60.7	61.4	54.2	54.0	5.5	5.1	0	0	8.1	8.2	5.3	5.0
**10**	15.9	16.2	15.3	15.1	49.4	48.8	6.2	6.5	0	0	6.9	6.7	5.0	5.3
**11**		17.4		85.1		82.3		5.6		0		7.4		6.1
**12**		15.0		58.5		64.7		6.1		0		7.9		5.6
**13**		16.8		58.3		57.6		5.2		7		7.6		6.0
**14**		13.9		77.5		42.0		5.3		0		6.9		6.1
**15**		14.1		53.7		84.3		6.2		11		7.3		6.2
**16**		14.7		82.6		70.8		5.8		0		7.8		5.7
**17**		15.1		11.3		86.7		6.2		11		7.3		6.7
**18**		16.3		11.7		69.6		7.2		0		7.9		6.4
**19**		17.4		73.1		52.1		6.0		5		8.0		6.3
**20**		14.6		14.7		64.4		4.8		0		7.8		6.6

LBC, Length of bone canal; ECSAC, External cross-sectional area of the canal; ICSAC, Internal cross-sectional area of the canal; MED, Middle ear depth; VAC, Vertical angle of canal; LM, Length of malleus; LI, Length of incus.

**Table 4:** Descriptive statistical measures related to the human ear.

	LBC (mm)	ECSAC (mm^2^)	ICSAC (mm^2^)	MED (mm)	VAC (°)	LM (mm)	LI (mm)
**Median**	15.00	64.00	63.65	6.20	0	7.60	6.05
**Interquartile range**	2.22	87.10	21.87	1.52	1.00	1.32	1.15
**Maximum**	17.40	31.20	86.7	7.5	11.00	8.7	6.8
**Minimum**	13.00	11.30	42.00	4.8	0	6.00	5.00

LBC, Length of Bone Canal; ECSAC, External Cross-Sectional Area of the Canal; ICSAC, Internal Cross-Sectional Area of the Canal; MED, Middle Ear Depth; VAC, Vertical Angle of Canal; LM, Length of malleus; LI, Length of incus.

The comparative analysis between the measurements of goat and human ears, with the value of the Mann-Whitney *U* test and the value of the statistical significance of each measurement are described in Table 5.

**Table 5:** Comparative analysis of goat and human ear measurements.

	LBC (mm)	ECSAC (mm^2^)	ICSAC (mm^2^)	MED (mm)	VAC (°)	LM (mm)	LI (mm)
**Mann-Whitney *U* Test**	426.00	688.00	305.00	897.50	0.00	500.00	600.00
**p**	0.72	0.00^a^	0.03	0.00^a^	0.00^a^	0.45	0.00^a^

LBC, Length of bone canal; ECSAC, External cross-sectional area of the canal; ICSAC, Internal cross-sectional area of the canal; MED, Middle ear depth; VAC, Vertical angle of canal; LM, Length of malleus; LI, Length of incus.

The length of the bone canal (15.10, 2.13 [12.30–19.90] vs. 15.00, 2.23 [13.00–17.40]; U = 426; *p* = 0.72) and the length of the malleus (7.35, 0.45 [6.90–7.90] vs. 7.60, 1.32 [6.00–8.70]; U = 500.00; *p* = 0.45) showed no statistically significant difference between goats and humans. The external cross-sectional area of ​​the bone canal (23.85, 2.27 [23.70–55.00] vs. 64.00; 87.10 [11.30–31.20]; U = 688.00, *p* = 0.00 ]; the internal cross-sectional area of ​​the canal (73.45, 18.65 [53.10–86.39] vs. 63.65; 21.87 [42.00–86.70); U = 305.00; *p* = 0.03]; the middle ear depth (4.20, 0.45 [3.20–4.90] vs. 6.20; 1.52 [4.80–7.50]; U = 897.50; *p* = 0.00)]; the vertical angle of the canal (44.50, 13.00 [27.00–65.00] vs. 0.00; 1.00 [0.00–11.00); U = 0.00, *p* = 0.00]; and the length of the incus (3.00, 0.27 (2.10–3.50) vs. 6.05; 1.15 (5.00–6, 80); U = 600.00, *p* = 0.00] showed a statistically significant difference between goats and humans.

**Figure 11:**
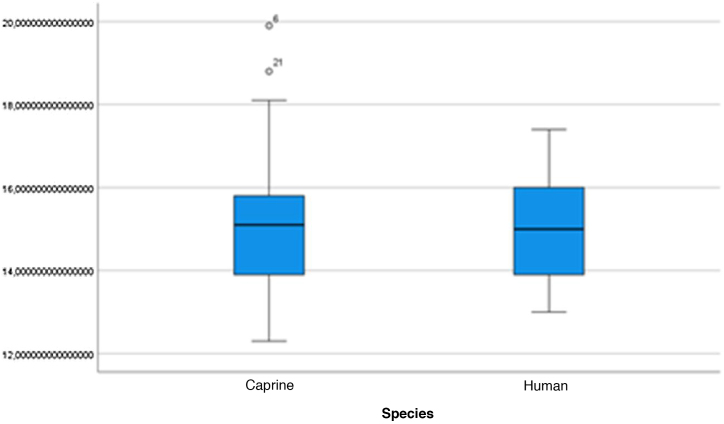
Length of the bone canal. Analysis of the measurements of goat and human bone canal length.

**Figure 12:**
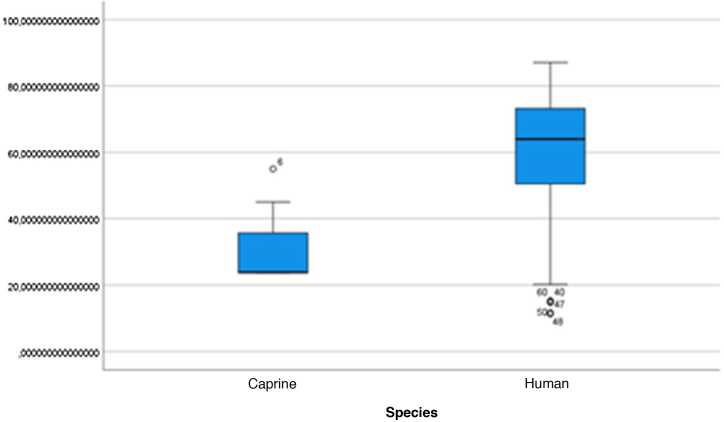
External cross-sectional area of the canal. Analysis of the measurements of the external cross-sectional area of goat and human canal.

**Figure 13:**
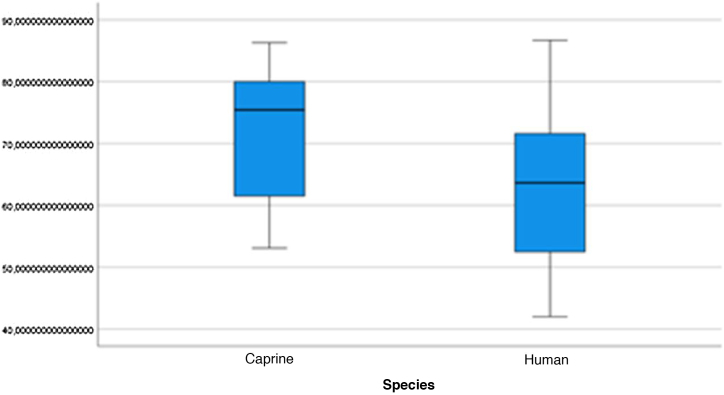
Internal cross-sectional area of the canal. Analysis of the measurements of the internal cross-sectional area of the goat and human canal.

**Figure 14:**
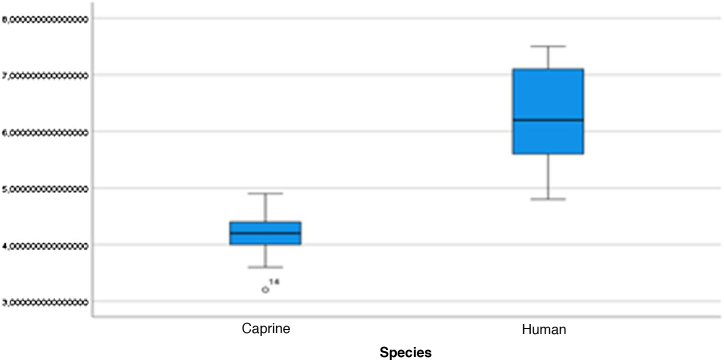
Middle ear depth. Analysis of the depth measurements of the goat and human middle ear.

**Figure 15:**
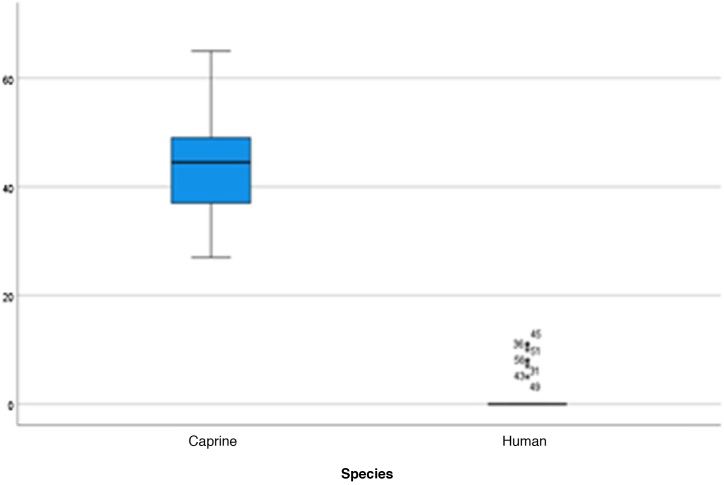
Vertical angle of the canal. Analysis of vertical angle measurements of goat and human canal.

**Figure 16:**
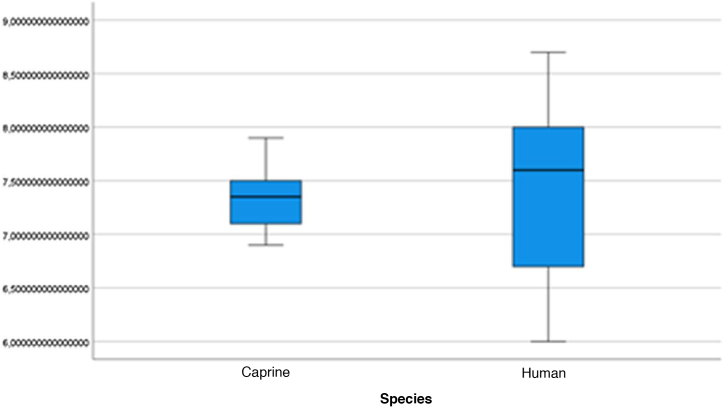
Malleus length. Analysis of goat and human malleus length measurements.

**Figure 17:**
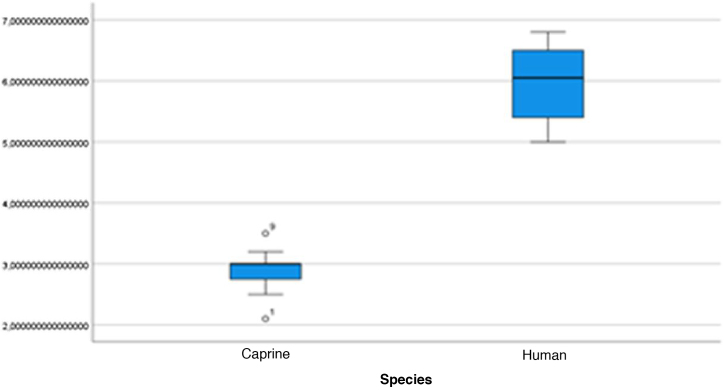
Incus length. Analysis of goat and human incus length measurements.

The graphical evaluation of the anatomical structure measurements of goats and humans is shown in Figs. 11 to 17 (Box-Plot graphs).

The difference between the actual VAC and the simulated VAC through the drilling of the goat canal floor is shown in Table 6. Table 7 shows the statistical analysis of these measurements.

**Table 6:** Difference between tVAC and sVAC with floor drilling simulation.

Animal	tVAC (°)		sVAC (°)		tVAC–sVAC (%)	
	RE	LE	RE	LE	RE	LE
1	55	51	5	8	−90.9	−84.3
2	45	47	10	10	−77.8	−72.5
3	49	44	25	23	−48.9	−47.7
4	47	47	29	30	−38.3	−36.2
5	35	37	10	10	−71.4	−54.0
6	30	31	4	1	−86.6	−96.8
7	30	27	20	17	−33.3	−37.0
8	37	39	15	19	−59.4	−51.3
9	44	47	28	32	−36.3	−31.9
10	69	66	25	26	−63.7	−60.6
11	53	50	26	24	−50.9	−52.0
12	36	39	15	15	58.3	−61.5
13	39	39	11	10	−71.8	−74.3
14	42	45	20	17	−52.4-	−62.2
15	58	54	17	21	−70.6	−61.1

tVAC, True vertical angle of the canal; sVAC, Simulated vertical angle of the canal.

**Table 7:** Statistical analysis of differences between tVAC and sVAC.

	tVAC	sVAC
**Mean**	44.40	17.43
**Standard deviation**	10.08	8.32
**Maximum**	69.00	32.00
**Minimum**	27.00	1.00

tVAC, True vertical angle of the canal; sVAC, Simulated vertical angle of the canal.

### Endoscopic evaluation

**Figure 18:**
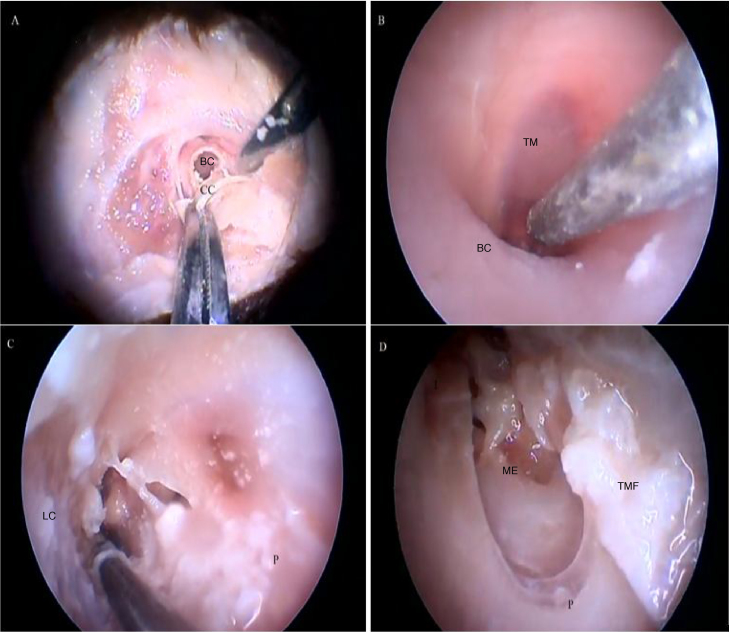
Steps performed in the endoscopic dissection. A, CC resection; B, macroscopic evaluation of BC and TM; C, LC drilling; D, preparation of the TMF and visualization of the ME. CM, Cartilaginous canal; BM, Bone canal; TM, Tympanic membrane; LC, Lower portion of the canal; P, Posterior canal; ME, Middle ear; TMF, Tympanomeatal flap.

Fig. 18 illustrates the steps performed in the endoscopic dissection.

**Figure 19:**
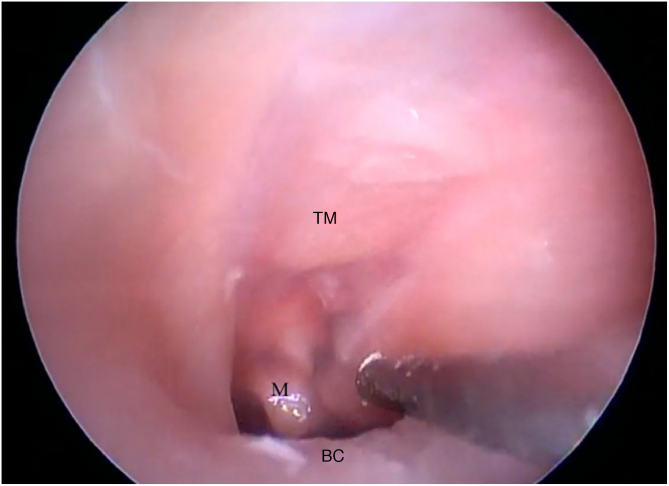
Bone canal and goat ear tympanic membrane. TM, Tympanic membrane; M, Malleus; BC, Bone canal.

The cartilaginous portion of the auditory canal is long and narrow and needs to be removed to advance the endoscope. The BC is also a narrow cavity, with a sharp downward curvature, preventing the visualization of the lower edge of the TM. This, in turn, has a light pink color, with its upper edge inserted more laterally. It is possible to visualize the malleus through the transparency of the TM (Fig. 19).

**Figure 20:**
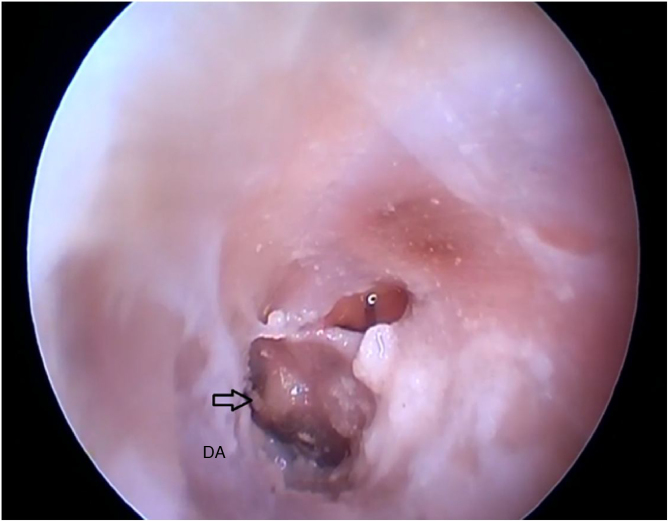
Lower edge of the tympanic membrane after drilling the lower portion of the canal. DA, Drilled area.

Fig. 20 shows the visualization of the TM after the drilling of the lower BC wall.

**Figure 21:**
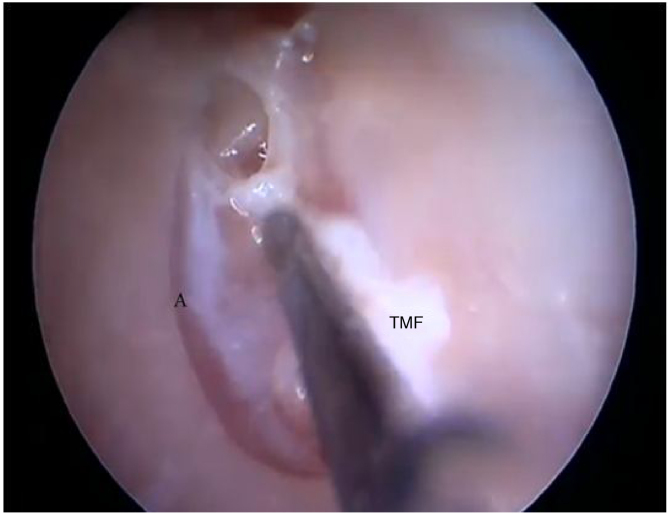
Visualization of the tympanic annulus after flap detachment. A, Annulus; TMF, Tympanomeatal flap.

Two incisions were made in the posterosuperior region of the BC, creating the tympanomeatal flap. With the detachment of this flap, it was possible to visualize a white, fibrous structure, corresponding to the tympanic annulus (Fig. 21). Due to the marked tortuosity of the BC, it was initially difficult to create the flap.

**Figure 22:**
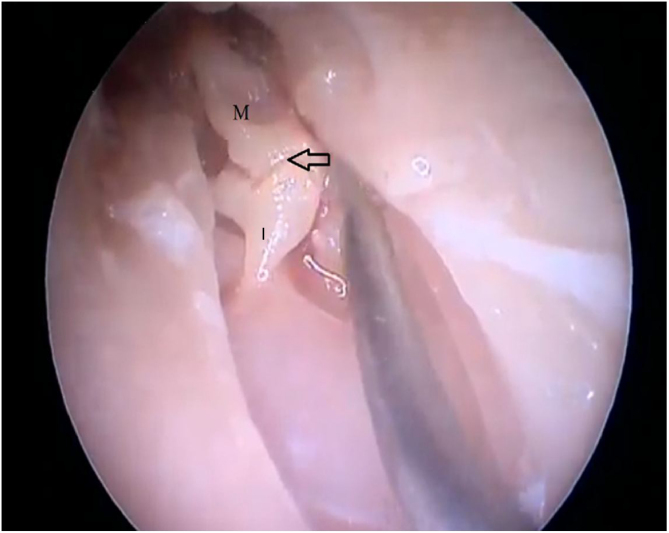
Visualization of the incudomalleolar joint. M, Malleus; I, Incus.

**Figure 23:**
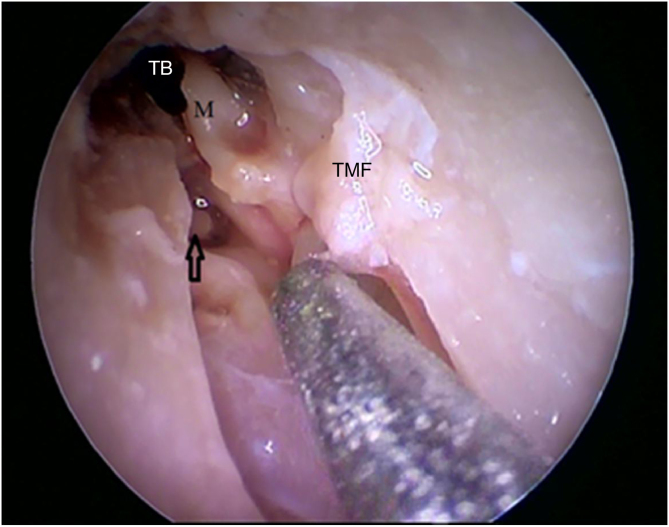
Visualization of the stapes after incus detachment (arrow). M, Malleus; TMF, Tympanomeatal flap; BT, Tympanic bulla.

**Figure 24:**
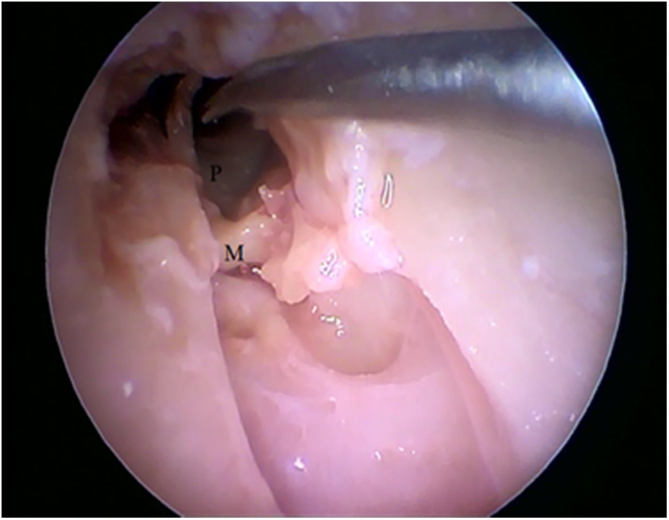
Detachment of the malleus with the identification of the promontory. P, Promontory; M, Malleus.

By exposing the tympanic cavity, the incudomalleolar joint was identified. After its detachment and removal of the incus, the stapes was visualized. The promontory and the tympanic bulla, corresponding to the hypotympanum, were identified after removal of the malleus (Figure 22, Figure 23, Figure 24). The facial nerve was not identified in the studied goat head.

## Discussion

Although there are differences between the measurements found in the ears of the animal model when compared to the human ear, the goat can be considered a viable model for the training of some steps of otological surgery, as well as the handling of an endoscope, while having a sense of the deep perception of the latter.

The exploratory analysis of the dispersion measures used, especially the median and interquartile range values, shows that the 15 goats had a homogeneous distribution of measurements.

The present study aimed at studying the viability of the goat model for the training of initial apprentices in endoscopic ear surgery, focusing on the handling of optics, instruments through the endaural route, as well as the training for less complex procedures, such as myringotomy, placement of a ventilation tube (VT) and the important steps of the performance of simple tympanoplasty, such as detachment of the tympanic-meatal flap, lifting of the tympanic annulus and positioning of the graft via the endaural route.

Ianacone et al. described that the ear canal of sheep was narrower than that of humans.^11^ The goat BC is also narrower and extremely tortuous. The lower edge of the tympanic membrane is inserted into a bony prominence, making it difficult to detach this membrane and study the tympanic cavity. In this case, for the adequate use of the goat model for dissection, it is important to remove this bony prominence, which can add to the development of the ability to drill into the external auditory canal, sometimes necessary in otologic surgeries.

In the endoscopic evaluation performed, the goat model was used, initially, to train the handling of optics and endaural surgical instruments. After that, with an appropriately sized drill, the caudal portion of the BC was enlarged, without removing the bone directly adjacent to the tympanic membrane, aiming to provide better access to the study of the middle ear. During a possible training in surgical skills, the more experienced the surgeon becomes, the less the need for enlargement of the external auditory canal.

Anschuetz et al. described during the study of the ovine model, that the external ear, which covers the ovine external canal, must be partially amputated to allow access to the EAC. This step was also performed during the present work with the goat model. Other similarities found between the two models were the presence of a bony prominence in the EAC, preventing access to the tympanic membrane, and a large pars flaccida in the tympanic membrane covering the epitympanic space. As a limitation of the ovine model, the authors mention the absence of a tympanic annulus, separating the tympanic membrane from the middle ear mucosa.^9^ As for the goat ear, all studied heads showed a tympanic annulus, an important structure in tympanomeatal flap detachment.

Zaid et al. performed a canalplasty in 8 goat models and, after this adaptation, developed a surgical training program for myringotomy, tympanoplasty with the use of cartilage graft, stapedectomy, facial nerve decompression and cochlear implant. Additionally, another advantage described in this study was the possibility to train the collection and preparation of cartilage from the goat's ear pinna, used in some otological surgical procedures.^10^

As the study sought to assess the feasibility of using a goat model for training in endoscopic surgeries, it is considered that the canal angulation problem is less important, since telescopes allow the angled vision to different degrees. Even the 0° telescope allows for expanded access, as it has an open angle of view. Thus, it is interesting to compare the measurements of VAC in goats with the angles of view of the most commonly used telescopes for endoscopic ear surgeries. These allow the visualization of structures that are displaced from their main axis from 45° (conventional straight view telescope) to 105° (45° wide angle telescope). By measuring the angles of the canal, it can be observed that a conventional 0° telescope would allow the view of the lower portion of the membrane in 16 (sixteen) of the 30 goat temporal bones, whereas the “wide angle” model would allow this view in 28 (twenty-eight). Any of the other telescopes (30° or 45°) would allow the viewing of all 30 goat temporal bones.

The VAC reduction presented in the results simulates what would be achieved with the drilling of the canal floor. And in this case, all angles would be within the 45° range of view of a conventional 0° telescope. However, as some angulation persists in many specimens, although it is possible to visualize all the elements at the bottom of the canal, not all of them would allow the handling of the structures with straight instruments, such as surgical drills. This brings a limitation regarding the possibility of drilling the juxta-tympanic portion of the bone protuberance that was observed in all animals. Also, the advance of the telescope towards the membrane could easily allow the use of the angled instruments conventionally used in otological surgery (detachers, micro-hooks, curved stylets, etc.), for handling all the tympanic margins.

An important factor to be considered is the geographic availability of the animal model that will be used in training, so that it becomes more accessible. In our territory and in many developing countries, goat farming has been increasing in recent years, making the goat a very low-cost surgical training model when compared to the sheep model.

The present study aimed to morphometrically describe the external and middle ear of goats, aiming to analyze the possibility of its use in the development of necessary skills when performing otological procedures such as myringotomy, VT placement and simple tympanoplasty. Despite some differences described in the results, the goat model can be useful in training the handling of the endoscope and microsurgical instruments, ensuring greater surgical skill and safety for its practice in humans.

As a limitation of the present study, the accurate identification of the lower edge of the tympanic membrane may be hindered by the presence of the juxta-tympanic protuberance, where this edge is inserted, found in all animals. The stapes was not visualized and the incus was not identified in ten goat temporal bones. However, veterinary anatomy textbooks have described that small ruminants have all three ossicles.^12^ It was also possible to visualize all three ossicles during the endoscopic evaluation. The non-identification of the stapes in the tomographic images may have occurred due to the thickness of the sections.

Subsequent studies of endoscopic training will help to understand the usefulness of the goat model as a means of acquiring skills for endoscopic otological surgery.

## Conclusion

The spatial configuration of goat ear structures and tissue consistency can allow the use of this model for the training of endoscopic otological surgical skills, related to the handling of instruments, the endoscope and steps of surgical techniques, such as tympanomeatal flap detachment.

## Conflicts of interest

The authors declare no conflicts of interest.

The authors of the manuscript entitled “Descriptive study of goat external and middle ear through computed tomography and endoscopic evaluation, compared with the human ear” declare that they have no financial, political, commercial, academic or personal conflicts of interest.
